# SIRT1 Prevents R-Loops during Chronological Aging by Modulating DNA Replication at rDNA Loci

**DOI:** 10.3390/cells12222630

**Published:** 2023-11-15

**Authors:** Bhushan L. Thakur, Nana A. Kusi, Sara Mosavarpour, Roger Zhu, Christophe E. Redon, Haiqing Fu, Anjali Dhall, Lorinc S. Pongor, Robin Sebastian, Fred E. Indig, Mirit I. Aladjem

**Affiliations:** 1 Developmental Therapeutics Branch, Center for Cancer Research, National Cancer Institute, National Institutes of Health, Bethesda, MD 20892, USA; bhushan.thakur@nih.gov (B.L.T.); nanaagyeman.kusi@nih.gov (N.A.K.); saramosavarpour@gmail.com (S.M.); liqunz803@gmail.com (R.Z.); redonc@mail.nih.gov (C.E.R.); fuha@mail.nih.gov (H.F.); anjali.dhall@nih.gov (A.D.); pongorlorinc@gmail.com (L.S.P.); robin.sebastian@nih.gov (R.S.); 2 Confocal Imaging Facility, National Institute on Aging, National Institutes of Health, Baltimore, MD 21224, USA; indigfr@grc.nia.nih.gov

**Keywords:** aging, cancer, chromatin, genomic stability

## Abstract

In metazoans, the largest sirtuin, SIRT1, is a nuclear protein implicated in epigenetic modifications, circadian signaling, DNA recombination, replication, and repair. Our previous studies have demonstrated that SIRT1 binds replication origins and inhibits replication initiation from a group of potential initiation sites (dormant origins). We studied the effects of aging and SIRT1 activity on replication origin usage and the incidence of transcription–replication collisions (creating R-loop structures) in adult human cells obtained at different time points during chronological aging and in cancer cells. In primary, untransformed cells, SIRT1 activity declined and the prevalence of R-loops rose with chronological aging. Both the reduction in SIRT1 activity and the increased abundance of R-loops were also observed during the passage of primary cells in culture. All cells, regardless of donor age or transformation status, reacted to the short-term, acute chemical inhibition of SIRT1 with the activation of excessive replication initiation events coincident with an increased prevalence of R-loops. However, cancer cells activated dormant replication origins, genome-wide, during long-term proliferation with mutated or depleted SIRT1, whereas, in primary cells, the aging-associated SIRT1-mediated activation of dormant origins was restricted to rDNA loci. These observations suggest that chronological aging and the associated decline in SIRT1 activity relax the regulatory networks that protect cells against excess replication and that the mechanisms protecting from replication–transcription collisions at the rDNA loci manifest as differentially enhanced sensitivities to SIRT1 decline and chronological aging.

## 1. Introduction

Aging is an inexorable biological process characterized by a decline in cellular function over time. A hallmark of aging is the accumulation of DNA damage arising from both intrinsic insults, i.e., reactive oxygen species, replication stress, and telomere dysfunction, and extrinsic insults, i.e., radiation and chemical exposures [1]. The activation of the DNA damage repair pathways in response to DNA damage leads to a loss of genetic information that also drives aging and induces senescence. This is often observed in survivors of childhood cancer, who exhibit premature aging and other kinds of sequelae, as the cytotoxic effects of chemotherapeutic treatments can induce DNA damage and impair genome stability [2]. Cellular senescence, a state of irreversible cell cycle arrest, is a fundamental aspect of the biology of aging and contributes significantly to the aging phenotype. Senescent cells undergo dramatic changes in gene expression related to chromatin remodeling and a persistent DNA damage response [3,4]. Such senescent cells accumulate in multiple tissues with time and can compromise tissue repair and regeneration through the depletion of progenitor cells, thereby contributing toward aging.

The ribosomal DNA (rDNA) theory of aging posits that instability in rDNA regions is a major driver of senescence [5]. The rDNA locus exhibits a highly conserved structure across eukaryotes, characterized by long tandem arrays of repeating units [6,7,8]. During interphase, rDNA arrays are located in the nucleolus and exhibit high transcriptional activity that is required for ribosome biogenesis. Markedly, yeast aging is characterized by rDNA instability, which is accompanied by the formation of extrachromosomal DNA circles (ERCs) derived from rDNA repeats. The formation of ERCs is associated with nucleolar fragmentation and enlargement. In yeast, the accumulation of ERCs over time exacerbates genomic instability, hastening cellular senescence and aging-related phenotypes [5]. ERCs are regulated by the yeast Sir2 protein, an NAD+-dependent histone deacetylase that has been found to extend the replicative lifespan of yeast cells by silencing the transcription of rDNA repeats [9].

SIRT1, one of the human nuclear orthologs of Sir2, has been implicated in various cellular processes, including DNA repair and apoptosis [10]. The SIRT1 expression in tissues decreases with age, and an increased SIRT1 expression has been shown to extend the lifespan of several organisms [11,12,13]. SIRT1 alters aging through its enzymatic activity by modifying the acetylation status of critical substrates to modify their cellular levels and/or enzymatic activities. Particularly salient in the context of aging and cellular senescence, SIRT1 plays a crucial role in the regulation of DNA replication origins, the genomic sites from which replication commences [14,15,16,17]. Not all potential replication origins are used during each cell cycle, and SIRT1 contributes to the selection and timing of which replication origins are activated in both yeast [18,19,20] and mammalian cells [21,22]. By deacetylating specific proteins involved in DNA replication, SIRT1 can regulate the activation of the pre-replicative complex (pre-RC), thus determining whether a particular replication origin will be used. The ability to prevent initiation from dormant origins is essential for genome stability and integrity [21,23,24,25].

The excessive initiation of DNA replication on transcribed chromatin can heighten the occurrence of collisions between advancing replication forks and transcription complexes, potentially forming three-strand RNA-DNA hybrids known as R-loops [23,24,25]. While R-loops play a crucial role in many cellular processes, their persistence at the rDNA locus contributes to genomic instability. As R-loops accumulate, they can induce DNA damage, disrupt DNA replication, and interfere with gene expression, all of which are hallmarks of cellular senescence and aging.

rDNA houses multiple replication origins, and only a portion of those origins are activated at distinct times during the S-phase of the cell cycle to initiate replication before each cell division [26,27]. We investigated if changes in SIRT1 activity led to the dysregulation of the balance between active and dormant origins and if such dysregulation can, in turn, trigger aberrant replication and genomic instability and contribute to cellular senescence. To this end, we studied the consequences of SIRT1 inhibition during chronological aging and asked if certain genomic regions are impacted differently by SIRT1. Here, we report the effects of chronological aging and SIRT1 activity on R-loop levels and replication origin usage in normal fibroblasts obtained at different time points during chronological aging, as well as in cancer cells. We observed that, in primary untransformed fibroblasts, SIRT1 activity declined, and the prevalence of R-loops rose with chronological aging, as well as with passage in culture. In cancer cells, the incidence of R-loops, as well as the frequency of replication initiation events, increased when SIRT1 activity declined. In contrast, in non-transformed adult fibroblasts, the decrease in SIRT1 activity and the increased prevalence of R-loops during chronological aging did not coincide with changes in genome-wide replication initiation profiles. Instead, the activation of dormant origins was observed within rDNA repeats. These observations suggest that the mechanisms protecting from replication–transcription collisions at the rDNA loci manifest as a distinct sensitivity to SIRT1 inhibition and chronological aging.

## 2. Materials and Methods

### 2.1. Cell Culture, Chemicals, PDL, and Establishment of Stable Cell Lines 

Normal human skin fibroblasts collected from two individuals, each on two occasions 15 years apart, were obtained from the Coriell Institute’s Aging Cell Depository. The fibroblast lines AG04441B and AG13156, collected from an individual donor at ages 29 and 44 years, and AG04446 and AG12851, collected from an individual donor at age 48 and 63, were grown at 37 °C in a 5% CO_2_ atmosphere in MEM (Thermo Fisher, Waltham, MA, USA, cat# 11095080) medium with 15% FBS. All four fibroblast cells were immortalized by human Telomerase Reverse Transcriptase protein (hTERT) stable expression using the hTERT Cell Immortalization Kit (ALSTEM, Richmond, CA, USA, cat# CILV02), as per the manufacturer’s instructions. The population doubling levels (PDL) were calculated as below:

PDL = 3.32 (log (total viable cells at harvest/total viable cells at seed))

Human HEK293, HCT116, MCF7, and U2OS cells were grown at 37 °C in a 5% CO_2_ atmosphere in RPMI medium (Thermo Fisher, 11875-119), supplemented with 10% FBS. All original cancer cell lines were obtained from ATCC (www.atcc.org (accessed on 26 September 2023)), and all cell lines were tested as negative for mycoplasmas (Lonza, Cambridge MA, USA, cat#LT07–418). The SIRT1 gene from all 4 cancer cell lines was deleted using CRISPR-Cas9 targeting exon1 and clones were confirmed using Sanger sequencing. EX527 (cat# E7034), 5-Bromo-2′-deoxyuridine, BrdU (cat# B5002), and thymidine (cat#T3763) were purchased from Sigma, St. Louis, MO, USA. On-target siRNA smart pool against SIRT1(L-003540-00-0005) was purchased from Dharmacon, Lafayette CO, USA.

### 2.2. R-Loop Slot Blot

For R-loop detection using the slot-blot procedure, genomic DNA was extracted from respective fibroblast cells using the DRIP protocol, as described previously [28]. Briefly, cells were lysed in 85 mM KCl, 5 mM PIPES (pH 8.0), and 0.5% NP-40 for 10 min on ice and centrifuged to isolate the nuclei. Pelleted nuclei were resuspended in lysis buffer (10 mM Tris-HCl pH 7.5, 200 mM NaCl, and 2.5 mM MgCl_2_ with 0.2% sodium deoxycholate, 0.1% SDS, and 0.2% Triton-X-100) and DNA: RNA were isolated using phenol/chloroform, ethanol precipitated, and resuspended in TE buffer. Five micrograms of genomic DNA was digested using a cocktail of restriction enzymes (HindIII, SspI, EcoRI, BsrGI, and XbaI; 15U each), and mRNA and other RNA were digested with RNase A (10 μg/mL; ThermoFisher Scientific, Waltham, MA, USA, cat# EN0531) and shortcut RNase III (2 units; New England Biolabs, Ipswich, MA, USA; Cat# M0245L) and re-purified via phenol/chloroform/isoamyl alcohol (25:24:1) extraction. In total, 200 ng of genomic DNA was spotted on a nitrocellulose membrane, crosslinked with UV light (120 mJ/cm^2^)), blocked with PBS-Tween (0.1%) buffer and 5% non-fat milk (Room temperature for 1 h), and incubated with mouse S9.6 antibody (1:500 dilution, overnight at 4 °C, (Millipore Sigma, Rockville, MD, USA, cat# MABE1095). After washing with PBS-Tween (0.1%), the membrane was incubated with HRP-conjugated anti-mouse secondary antibody, washed, and signals were captured using a BioRad imager.

### 2.3. R-Loop Immunofluorescence Analysis

Fibroblast cells were cultured on a cover glass and treated with and without EX527 (1 μM for 48 h). The cells were pulse-labeled with 10 μM EdU for 60 min before harvest. For staining, cells on a glass surface were incubated in PBS-T buffer (0.2% Triton X-100 in 1  ×  PBS, phenylmethylsulphonylfluoride (PMSF), a protease inhibitor cocktail (Sigma, P8340), and phosphatase inhibitor cocktail (Roche, San Francisco, CA, USA, cat# P4906845001) for 5 min on ice, followed by fixation with 2% paraformaldehyde. EdU staining was performed using the Click-iT EdU kit (ThermoFisher Scientific, Waltham, MA, USA, cat# C10634 (for AL647). Primary antibody staining using mouse S9.6 antibody (1:200 dilution) and rabbit nucleolin (1:250 dilution) was performed for 3 h at room temperature. Secondary antibody staining was performed as follows: Alexa 488-conjugated anti-mouse immunoglobulin G (IgG), IgG2b, Alexa 568-conjugated anti-rabbit IgG, and Alexa 647-conjugated anti-human IgG (1:500, ThermoFisher Scientific, Waltham, MA, USA, cat#A11029 and cat# A21445) for 1 h at room temperature.

### 2.4. Flow Cytometry 

The cells were pulse-labeled with 10 μM EdU for 60 min before harvest. EdU staining was performed using the Click-iT EdU kit (ThermoFisher Scientific, Waltham, MA, USA, cat# C10634 (for AL647) or cat# C10633 (AL488)) according to the manufacturer’s protocol. For senescence detection, the CellEvent™ Senescence Green Flow Cytometry Assay Kit (ThermoFisher Scientific, Waltham, MA, USA, cat# C10841) was used, as per the manufacturer’s instructions. A BD LSR Fortessa cell analyzer with the FACSDiva software and/or FlowJo10.6 was used for cell cycle analyses.

### 2.5. SIRT1 Deacetylase Activity 

SIRT1-specific activity was estimated from the total cell extracts (in NP40 buffer) using a SIRT1 deacetylase activity kit (Abcam Waltham, MA, USA, cat# ab156065), as per the manufacturer’s instructions. Trichostatin A (20 nM) was added to lysate to block HDAC class I and II activity.

### 2.6. DNA Replication Analysis through Molecular Combing

An analysis of DNA replication through molecular combing was performed, as previously described [29]. Briefly, asynchronous cells were sequentially labeled with 20 μM CIdU for 30 min and 20 μM ldU for 30 min, then chased with 100 μM thymidine for 60 min. The cells were embedded in low-melting agarose plugs and long genomic DNA fibers were isolated and combed onto salinized coverslips (Genomic Vision, Nanterre, France, cat# cov-002-RUO) using an in-house combing machine. IdU, CldU, and single-stranded DNA were detected using a mouse antibody directed against BrdU (IgG1, Becton Dickinson, Washington, DC, cat# 347580, 1:25 dilution), a rat antibody directed against BrdU (Accurate chemical, Carle place, NY, USA, cat# OBT0030, 1:200 dilution), and a mouse antibody directed against single-stranded DNA (ssDNA) (IgG 2a, Millipore Sigma, Rockville, MD, USA, MAB3034, 1:100), respectively. The secondary antibodies used were goat anti-mouse Cy3 (Abcam ab6946), goat anti-rat Cy5 (Abcam, ab6565), and goat anti-mouse BV480 (Jackson ImmunoResearch, West Grove, PA, USA, cat# 115-685-166) for ssDNA. Slides were scanned with a FiberVision Automated Scanner (Genomic Vision). Replication signals on single DNA fibers were analyzed using FiberStudio (Genomic Vision, Nanterre, France). Only replication signals from high-quality ssDNA (not those from DNA bundles nor those located at the end of a strand) were selected for analyses.

### 2.7. Nascent Strand DNA Sequencing (NS-Seq)

Fibroblast lines, immortalized fibroblasts, and U2OS cells (harboring WT, KO, or H363Y SIRT1) without or with (EX527) treatment were harvested and genomic DNA was purified, with nascent strands being isolated as described previously [30,31]. Briefly, the DNA was denatured by boiling for 10 min, immediately cooled on ice, and fractionated on a neutral sucrose gradient. Fragments of 0.5–2 kb (containing nascent strand DNA and broken genomic DNA) were collected and treated with λ exonuclease to remove non-RNA-primed broken genomic DNA. The remaining single-stranded nascent strand DNA was converted into double-stranded DNA using the BioPrime DNA Labelling System (ThermoFisher Scientific, Waltham, MA, USA, cat# 18094011). Double-stranded nascent DNA (1 μg) was sequenced using the Illumina genome analyzer II (Solexa, Hayward, CA, USA). Sheared genomic DNA was also sequenced to be used for peak calling.

### 2.8. Chromatin-, DNA:RNA- Immunoprecipitation and Replication Specific R-Loop Mapping and Sequencing

ChIP-seq for pSIRT1 and pMCM2 was performed, as described earlier [21]. Briefly, U2OS cells were synchronized at the G1/S boundary (a double-thymidine block: 2.5 mM thymidine for 18 h followed by a release into fresh media for 9 h and 2.5 mM thymidine for 16 h). Fibroblast cells were serum-starved 24 h before double thymidine block. The G1/S synchronized cells were crosslinked with 1% formaldehyde for 10 min at RT and the remaining formaldehyde was quenched with glycine (1.25 mM) and washed twice with PBS. The cell nuclei were isolated using cytoplasmic extraction buffer with 0.25% NP40 buffer (volume of 5 times the pellet size or 500 μL, whichever was higher) plus proteinase and phosphatase inhibitor (1×) and incubated on ice for 5 min. The nuclei were collected through centrifugation at 2700× *g* and resuspended in 500 μL of NP40 buffer followed by sonication (40% aptitude, 1 s pulse, 65–80 pulses). The supernatants (from about 2 × 10^6^ cells) were precleared with protein G beads and incubated with phospho-MCM2 (S139) (Cell Signaling, Danvers, MA, USA, cat# 12958) or pT530-SIRT1 (in-house antibody) antibodies along with 80 µL of protein G beads overnight at 4 °C. The beads were washed twice with each of the following buffers: low-salt buffer, high-salt buffer, lithium chloride buffer, and TE (each spin at 1000× *g* for 1–2 min). The samples were eluted, incubated at 65 °C overnight for reverse-crosslinking, and then purified using a Monarch PCR & DNA Cleanup Kit (NEBT1030S). In total, 10 ng of ChIP samples were used to generate the library. The kits used for the library were the NEBNext Ultra II DNA Library Prep Kit for Illumina (New England Biolabs, Ipswich, MA, USA, cat# E7805S) and NEBNext Multiplex Oligos for Illumina (Index Primers Set 1 and Set 2) (NEB, E7335S and E7500S). Sequencing was conducted using the Illumina NextSeq 75 cycle High Output kit.

For R-loop mapping, genomic DNA was extracted from fibroblast cells using the DRIP protocol, as described previously [28]. Briefly, the cells were lysed in 85 mM KCl, 5 mM PIPES (pH 8.0), and 0.5% NP-40 for 10 min on ice and centrifuged to isolate the nuclei. Pelleted nuclei were resuspended in lysis buffer (10 mM Tris-HCl pH 7.5, 200 mM NaCl, and 2.5 mM MgCl_2_ with 0.2% sodium deoxycholate, 0.1% SDS, and 0.2% Triton-X-100) and DNA:RNA was isolated using phenol/chloroform, ethanol precipitated, and resuspended in TE buffer. In total, 5 μg of genomic DNA was digested using a cocktail of restriction enzymes (HindIII, SspI, EcoRI, BsrGI, and XbaI; 15U each), and mRNA and other RNA were digested with RNase A (10 μg/mL; ThermoFisher Scientific Cat# EN0531) and shortcut RNase III (2 units; New England Biolabs, Ipswich, MA, USA, cat# M0245L) and re-purified via phenol/chloroform/isoamyl alcohol (25:24:1) extraction. In total, 300 ng of genomic DNA was immunoprecipitated with mouse S9.6 antibody and processed similarly to the ChIP-seq described above.

For replication-specific R-loops mapping, BrdU was pre-incubated in fibroblast cells for 1 h before cell harvesting. The samples were processed for the S9.6 IP described above. Five different preparations were pooled, and then isolated DNA was used for the second IP with BrdU antibody. The samples were processed similarly to ChIP DNA isolation and the DNA was isolated in 50 μL, while 5 μL of isolated DNA was used for qPCR using primers specific to rDNA regions.

### 2.9. Nascent Strand and ChIP-Sequencing Analysis

Both NS-seq and ChIP-seq alignment, peak calling, and a coverage analysis were performed, as described earlier [32], except raw reads were aligned to the T2T (CHM13_v2) genome assembly [33]. Dormant origin locations were generated by subtracting baseline (WT) origins from the origins activated at SIRT1-depleted cells using the ‘bedtools subtract’ module (bedtools v2.30). To compare samples by coverage, the ‘BAMscale cov’(v0.0.6) method was prepared with either merged ChIP-seq regions (MACS narrowpeaks) or with consensus nascent-strand regions (MACS broad peaks) between comparative samples and alignment files for each sample. Regions were assigned normalized coverage values based on the library size normalization method of BAMscale. Peak density plots comparing sample pairs were created using R (v3.6); the code is available on the BAMscale GitHub page (https://github.com/ncbi/BAMscale/ (accessed on 26 September 2023)). For viewing in the IGV (Integrative genomics viewer), the BAMscale ‘scale–smallest’ method was used to prepare scaled bigWig coverage tracks for each alignment file in the set.

## 3. Results

To investigate whether R-loop abundance was affected by chronological aging during adulthood, we examined the R-loop prevalence, SIRT1 activity, and cell cycle characteristics in fibroblasts obtained from two individuals at 15-year intervals. The samples obtained from the Coriell Institute Aging Cell depository were taken from individual A at ages 29 and 44, and from individual B at ages 48 and 63 (see Supplementary Figure S1A for sample characterization). During early passages, 20–28% of cells were in the S-phase (Supplementary Figure S1B). Fibroblast cultures were passaged until senescence was detected as the absence of proliferation and an increased β-galactosidase activity (Supplementary Figure S1A,C). In parallel, cells at early passages were transfected with a lentivirus which stably expresses human Telomerase Reverse Transcriptase protein (hTERT) to create immortalized cell lines. In non-transformed fibroblasts, SIRT1 activity decreased in the later samples taken from both individuals, as well as with passaging (compare A29 and 44, B48 and 63; Supplementary Figure S1D). In later passages, when the cells reached senescence, a decrease in SIRT1 activity was observed in the samples from individual A and the younger sample from individual B. In the cells taken from 63-year-old individual B, the SIRT1 activity was very low, and we did not observe a further decrease. Fibroblast immortalization retained SIRT1 activity levels for at least 50 population doublings (Supplementary Figure S1E). 

**Figure 1:**
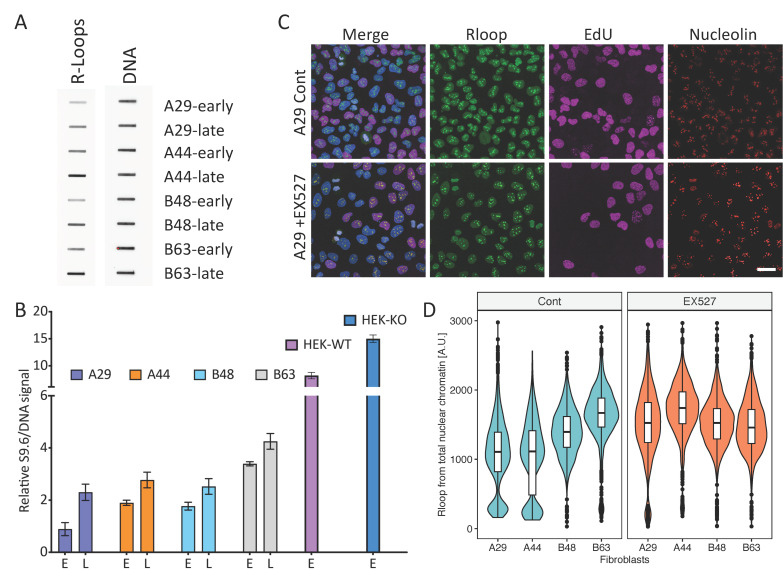
The prevalence of nuclear R-loops increased during chronological aging. (**A**,**B**): R-loop prevalence increased during chronological aging. Fibroblasts were cultured until senescence was detected, as described in Supplementary Figure S1A. R-loop levels during early passage and in pre-senescent (late) non-transformed fibroblasts were measured using the slot-blot protocol. Cells were lysed, nuclei were isolated, and DNA:RNA hybrids were slot-blotted and immunodetected using the S9.6 antibody. Signals were quantified using a Bio-Rad imager. Double-stranded DNA was used as a loading control. In A, a representative slot-blot; in B, a bar plot showing increased R-loop abundance in fibroblasts from an early passage (E) and a late passage, reaching senescence (L). Three independent R-loop slot blots were used. R-loop abundance was also measured in chromatin isolated from HEK293 cells harboring intact SIRT1 (HEK-WT) or in HEK293 cells in which SIRT1 was depleted (HEK-KO). (**C**,**D**): Immunofluorescence-based detection of R-loops measuring their abundance in nuclear chromatin (see Supplementary Figure S2A,B for nucleoplasm and nucleolar signal quantification). Fibroblasts were treated with 1 μM of the SIRT1 inhibitor EX527 for 48 h to inhibit the activity of SIRT1, or with DMSO as a control, and then pulse-labeled with 10 μM of EdU for 60 min. EdU-labeled DNA was visualized using a Click-iT EdU kit. In C, representative images from control and EX527-treated fibroblasts obtained from individual A at age 29 (A29). Scale bar = 20 μm. In (**D**), violin plots illustrating R-loop levels in human fibroblast cells taken from two individuals at different time points during chronological aging, treated with and without EX527.

We first asked if biological aging and the passage of cells in culture affected the prevalence of R-loops. R-loops detected on chromatin were more abundant in fibroblasts from older individuals, and the abundance of R-loops was increased in the fibroblasts reaching senescence (Figure 1A,B). Immunofluorescence-based detection clearly indicated R-loop presence in the nucleoli, as well as the nucleoplasm (Figure 1C). The abundance of R-loops within and outside the nucleolus was similar in the two samples obtained from individual A, but markedly increased with aging in individual B (Figure 1D and Figure S2A,B), along with the observed reduction in SIRT1 activity (Supplementary Figure S1D). In fibroblasts with active SIRT1 (derived from individual A and the earlier sample from individual B), the abundance of nuclear and nucleolar R-loops increased after exposure to SIRT1 inhibitor EX527 [34] (Figure 1D and Figure S2A). The inhibition of SIRT1 in the later sample from individual B (B63) lowered the abundance of R-loops to a level similar to the abundance observed after SIRT1 inhibition in the sample obtained 15 years earlier (B48). We did not observe significant changes in the R-loop abundance in non-nucleolar chromatin (Supplementary Figure S2B).

**Figure 2:**
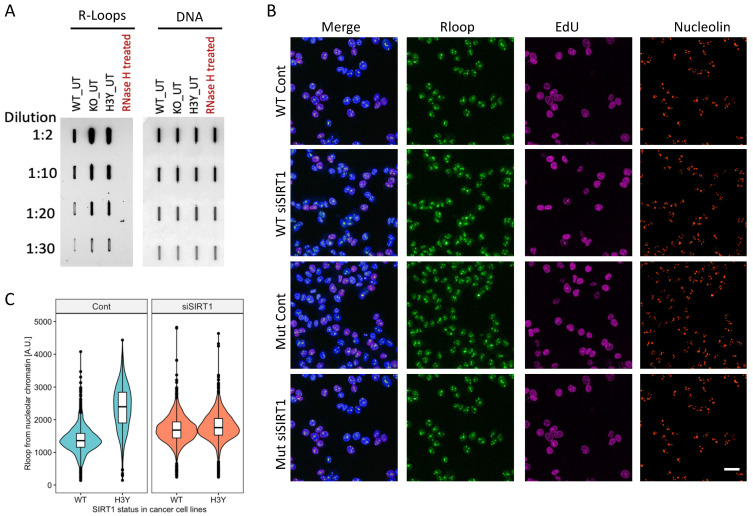
SIRT1 inhibition increased R-loops in cancer cells. (**A**): R-loop levels in U2OS cells harboring intact SIRT1 (WT), SIRT1 depleted (KO), and KO cells harboring a mutated (H363Y) SIRT1 (H3Y/Mut) measured using a slot blot, as described in the legend to Figure 1A. (**B**): Immunofluorescence-based detection of elevated R-loop prevalence upon SIRT1 loss. Immunofluorescence was detected and quantified, as described in the legend to Figure 1C. Scale bar = 20 μm. (**C**): Violin plots measuring R-loop prevalence in cells with mutated SIRT1.

Given the role of SIRT1 in dormant replication origin maintenance [21,22], we asked if SIRT1 activity lowered the incidence of R-loops by increasing replication origin dormancy, which, in turn, prevented transcription–replication collisions. To that end, we measured the prevalence of R-loops in an isogenic system of cancer cells deficient or proficient in SIRT1, and in U2OS cells harboring an inactive SIRT1 mutant (H363Y, henceforth referred to as H3Y/Mut) [21]. Our data showed that the absence of SIRT1 activity coincided with the increased prevalence of R-loops (Figure 2A–C and Figure S2C,D). In SIRT1-proficient cells, we also noticed a marked increase in the prevalence of R-loops upon a loss of SIRT1 and an even higher prevalence of R-loops in cells with mutated SIRT1 (Figure 2C and Figure S2C,D). The acute depletion of SIRT1 increased the abundance of R-loops in WT cells but not in Mut cells.

**Figure 3:**
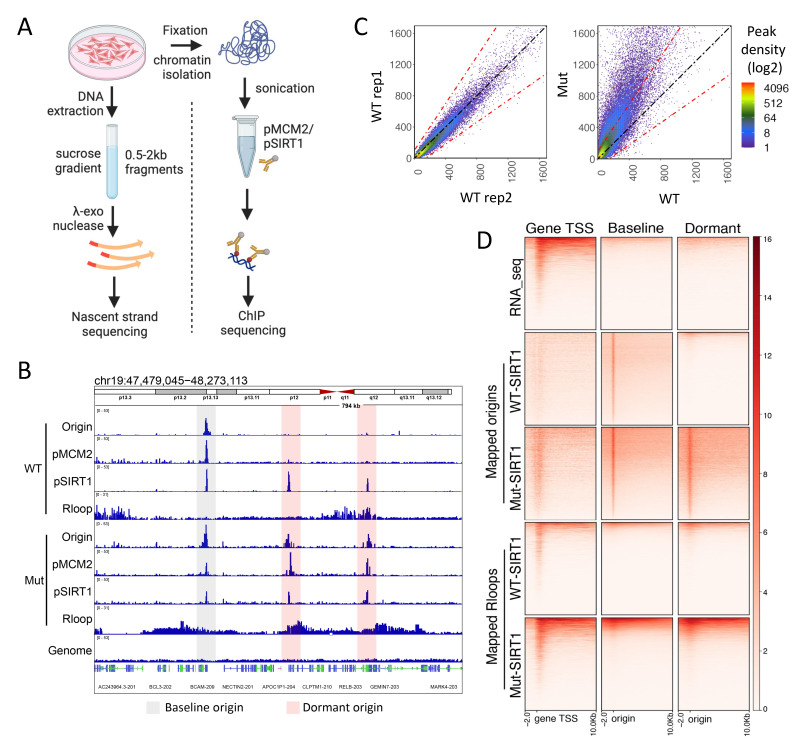
Loss of SIRT1 activity induces excessive origin activation and R-loops. (**A**): SIRT1-depleted U2OS cells harboring flagged versions of either intact (WT) or H363Y-mutated SIRT1 (Mut) were used for nascent strand sequencing (NS-seq) to quantify the activity of replication origins, chromatin immunoprecipitation sequencing (ChIP-seq) to map pMCM2-S139 and pSIRT1-T530 binding sites on chromatin, or immunoprecipitation with S9.6 to map the chromatin locations of R-loops. NS-seq and R-loop mapping were performed using unsynchronized cells, whereas pSIRT1 and pMCM2-S139 binding sites were mapped in G1/S synchronized cells. (**B**): A representative Integrated Genome Viewer (IGV) snapshot mapping replication initiation sites, pSIRT1 and pMCM2 binding sites on chromatin, and locations of R-loops in isogenic cells with and without SIRT1 activity. Grey highlights baseline origins (active in WT cells), whereas red highlights dormant origins, which are active in Mut cells only. (**C**): Scatterplot showing origin activity in WT and Mut-SIRT1 cells. Left, initiation activities of replication origins measured in WT cells—two independent replicates are compared. A diagonal black dotted line indicates equivalent origin initiation in comparative conditions, while red dotted lines indicate a 1.5-fold change in origin activity. Right, initiation activities of replication origins were measured in WT cells (*x*-axis) and Mut cells (*y*-axis). (**D**): A heatmap illustrating the locations and activity of transcribed genes, baseline origins, dormant origins, and R-loops in cells harboring WT and Mut SIRT1.

Next, we tested the hypothesis that SIRT1 diminished R-loops’ occurrence by attenuating replication stress, reflecting SIRT1-mediated suppressed initiation from dormant replication origins [21,22]. We first mapped the sites of replication initiation (replication origins), chromatin localization of SIRT1 binding sites, and R-loops in isogenic cancer cells proficient and deficient in SIRT1 activity (Figure 3A,B). Along with the activation of dormant origins, R-loop abundance was also increased in cells harboring mutant SIRT1. R-loops were mapped to regions that contained both baseline and dormant origins (Figure 3A–C and Figure S3A,B). Similar to origins, SIRT1 and phospho-MCM2 S139 binding sites overlapped with the majority of R-loop sites (Supplementary Figure S3C,D). Notably, although the locations of R-loops were not restricted to replication origins, both replication origins and R-loops were enriched in moderately transcribed regions (Figure 3D). In the absence of SIRT1, R-loop sites overlapped with regions in which dormant origins were activated (Figure 3D and Figure S3E), most likely due to replication–transcription conflicts. 

**Figure 4:**
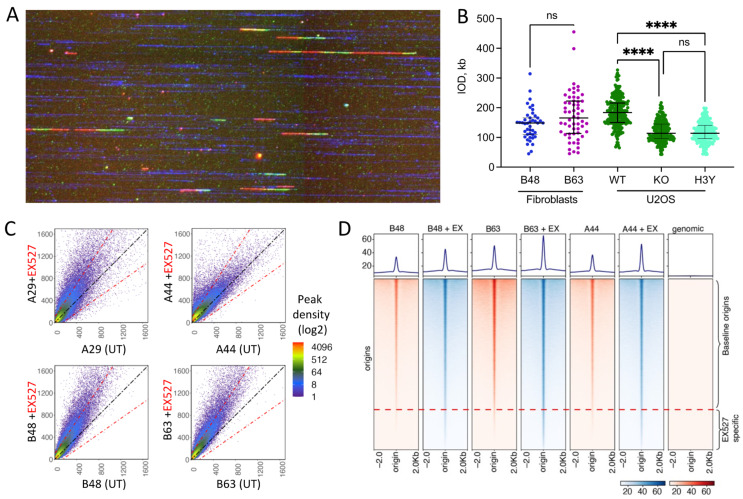
Replication origin activity and R-loop prevalence during aging. (**A**,**B**): Chronological aging did not alter inter-origin distances. In A, asynchronous fibroblast cells were labeled sequentially with CldU and IdU, followed by thymidine chase. Genomic DNA was embedded in low-melting agarose plugs, and long DNA fibers were combed on coverslips. Replication signals were detected using antibodies against BrdU and single-stranded DNA. Replication signals from high-quality ssDNA were selected for analysis using FiberStudio. In B, inter-origin distances (IOD) in DNA fibers from fibroblasts obtained from individual B at ages 48 and 63 and U2OS cells with active SIRT1 (WT), SIRT1 depleted (KO), or inactive SIRT1 (H3Y). Cancer cells with SIRT1 deficiency or inactive SIRT1 showed reduced inter-origin distances, indicating activation of dormant origins, as reported earlier [21]. No change in inter-origin distances indicate genome-wide utilization of dormant origins remained stable during this 15-year interval and cancer cells. Statistical significance was tested using Mann-Whitney test, **** indicates *p* < 0.0001, ns indicates no significance. (**C**): Peak density plots comparing replication origin usage between untreated (UT) and EX527-treated normal fibroblast samples obtained at different points during chronological aging. Activation of dormant origins was observed upon treatment with EX527. A diagonal black dotted line indicates equivalent origin initiation in comparative conditions and red dotted lines indicate a change in origin activity by 1.5 fold. (**D**): Heatmaps showing a population of replication origins activated upon the inhibition of SIRT1 with EX527. Ex = EX527 treatment.

The high prevalence of R-loops in SIRT1-deficient cells and the association of R-loops with replication origins raised the question of whether the increase in R-loop abundance during aging reflected a change in replication initiation profiles. Therefore, we probed into origin usage during chronological aging by assessing the replication origin activity in the primary fibroblasts obtained from individuals at 15-year intervals. A single-fiber analysis of the DNA replication patterns in the samples taken from individual B at ages 44 and 63 (Supplementary Figure S1A) showed that replication fork progression was slightly faster in fibroblasts taken at the older time point, but inter-origin distances were similar (Figure 4A,B and Figure S4A). Hence, replication origin utilization did not notably change during chronological aging. These analyses suggested that, although SIRT1 inhibition increased the frequency of initiation concomitant with the enhanced prevalence of R-loops in cancer cells, age progression did not trigger additional replication initiation events in adult fibroblasts. Then, we mapped the locations of replication initiation events using nascent strand sequencing (NS-Seq) in the matched pairs of primary fibroblasts and in fibroblasts exposed to the SIRT1 inhibitor EX527 [34]. SIRT1 inhibition increased the frequency of initiation (Figure 4C) and triggered initiation from a group of origins that were dormant in untreated cells (Figure 4D), similar to the activation observed in cancer cells [21]. In contrast, global replication origin usage and the relative frequency of replication initiation were largely similar in fibroblasts obtained from the same individual 15 years apart (Figure 4C,D and Figure S4B–E), despite the aging-related decline in SIRT1 activity. 

**Figure 5:**
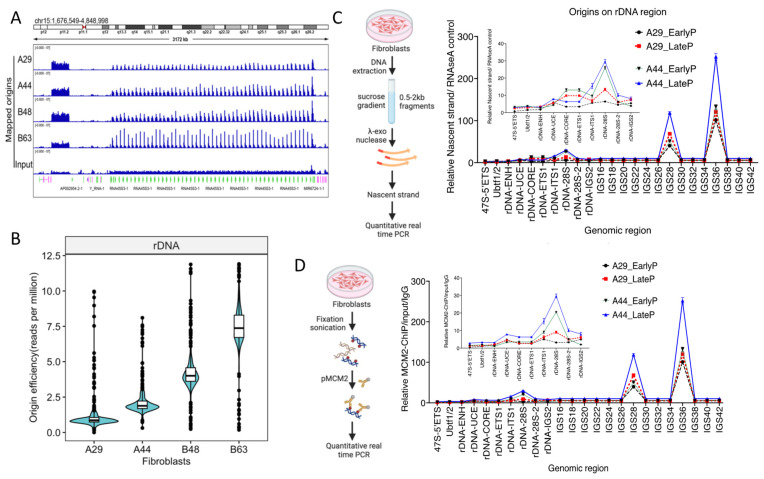
R-loop Prevalence at rDNA loci. (**A**,**B**): Increased replication initiation events at rDNA loci during chronological aging and in response to SIRT1 inhibition. (**A**): IGV screenshot showing NS-seq signals from four fibroblast cells at chromosome 15 rDNA repeats. (**B**): Violin plots quantifying replication initiation events at rDNA loci in the samples used in panel A. (**C**): Samples obtained at a later time point exhibited initiation of DNA replication at a site that was inactive in earlier samples, indicating the activation of a dormant origin. On the left, schematics of origin were mapped using nascent strand isolation followed by quantitative PCR. Isolated nascent strand DNA was quantified using real-time PCR using specific primers to the rDNA regions. On the right, a line plot showing the quantification of qPCR. Inset demonstrates qPCR quantification at the rDNA coding region, showing dormant origin in older age fibroblast and late passage cells. (**D**): On the left, a schematic of pMCM2 ChIP followed by qPCR at the rDNA unit. On the right is the quantification of pMCM2 ChIP using qPCR. Similar to NS-qPCR, pMCM2 confirmed the presence of baseline (non-coding region) and dormant (coding region) origins. Inset is quantification over the rDNA coding region.

Because the observed R-loops were highly abundant in nucleoli, we asked if chronological aging correlated with altered replication initiation patterns at the nucleolar-associated rDNA loci. Due to the high copy number of the rDNA loci, chromatin transactions, including replication initiation, R-loop loci, and protein binding, can be analyzed using sequencing and alignment to the telomere-to-telomere build of the human genome [33] and also quantified with high precision using quantitative PCR. We therefore measured replication initiation events at rDNA arrays in fibroblasts using both q-PCR and NS-Seq. These analyses showed that, although the overall replication patterns were not affected by the loss of SIRT1 activity during chronological aging (Figure 4C,D), replication initiation events at rDNA loci were more frequent with chronological aging (Figure 5A–C). Initiation frequency was further enhanced in cells treated with the SIRT1 inhibitor (Supplementary Figure S5B). Notably, we observed that the samples that were acquired at the later time point exhibited the initiation of DNA replication at a site that was inactive in the samples taken at the earlier time point, suggesting an activation of a dormant origin (A44; Figure 5C). Increased origin activity during chronological aging was observed in all five chromosomal locations of rDNA repeats (Supplementary Figure S5A). These observations support an active role of SIRT1 in the suppression of dormant origins at the rDNA region. We also detected the DDK-phosphorylated form of the MCM helicase (MCM2-S139) at a novel location in the older individual (Figure 5D), suggesting that, in the older individual, a pre-replication complex was assembled and activated at the dormant origin.

**Figure 6:**
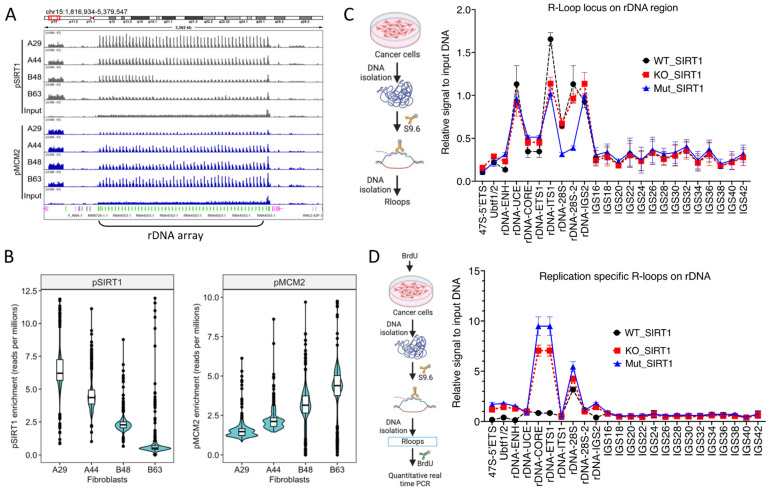
SIRT1 suppresses replication-specific R-loops at rDNA loci. (**A**): IGV screenshot depicting SIRT1 and phosphorylated MCM2 binding at rDNA loci in fibroblast cells from aging individuals. ChIP-seq for pSIRT1 and pMCM2 was performed as described in Figure 3 legend. (**B**): Quantification of pSIRT1 and pMCM2 binding. With aging, fibroblast cells exhibited reduced SIRT1 binding and enhanced phosphorylated MCM2 association at rDNA loci. (**C**): Left, schematics of R-loop measurement from cancer cells and right quantification of R-loop signal over rDNA region using qPCR. Increased R-loop prevalence at rDNA loci in cancer cells deficient in SIRT1 activity. (**D**): Left, schematics of detection of replication-specific R-loops from cancer cells. Quantification of R-loops was associated with newly replicated DNA showing increased replication-specific R-loop at the 28S rDNA gene promoter in SIRT1 inactive cells.

To evaluate directly if replication origin dormancy was associated with SIRT1 binding, we mapped SIRT1 binding sites and phospho-MCM2 binding sites at the rDNA loci in the fibroblast cells taken from individuals A and B, 15 years apart. Both SIRT1 and phosphorylated MCM2 (serine 139) bound replication origins at the rDNA loci. SIRT1 binding at putative dormant replication origins decreased in the samples taken from aging individuals, whereas the association with phosphorylated MCM2 increased (Figure 6A,B). Consistent with this, in cancer cells, although the total levels of R-loops in the rDNA coding region were not affected by SIRT1 activity, a loss of SIRT1 activity exhibited an increased frequency of replication-specific R-loops (Figure 6C,D). Specifically, we observed novel R-loops associated with newly replicated DNA (Figure 6D) at the promoter of the 28S rDNA gene. These R-loops were not detected in cells with intact (WT-)SIRT1 activity.

Finally, we asked if the modulation of SIRT1 activity during aging is associated with the altered expression of the rDNA arrays. In both the cancer cells and fibroblasts, the initiation of DNA replication at the G1/S boundary coincided with the inhibition of transcription at the rDNA loci, with a higher inhibition of transcription in the proliferating cells derived from younger individuals (Supplementary Figure S6A–E). These observations suggested that SIRT1 activity prevented the activation of a distinct origin at an earlier age, consistent with the presence of a dormant origin at the rDNA genomic region.

## 4. Discussion

**Figure 7:**
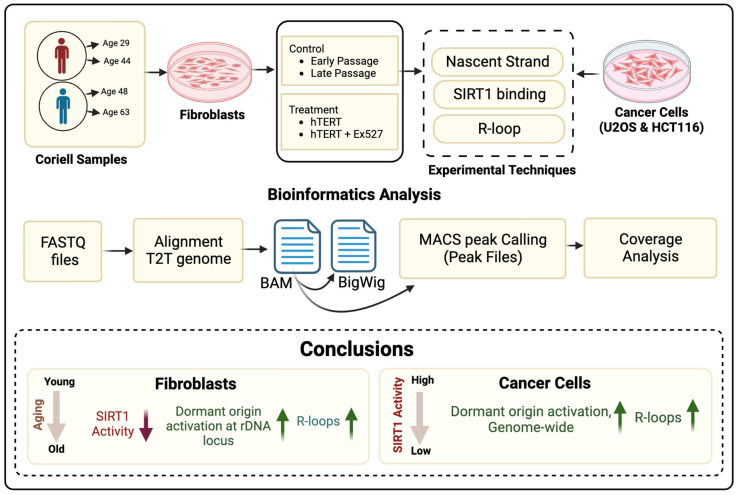
Summary of the impact of aging and loss of SIRT1 activity.

We report that primary human fibroblasts obtained from adults exhibited a decline in SIRT1 activity within 15-year intervals, correlating with earlier senescence in cells obtained from older individuals and an increased prevalence of R-loops. A similar decrease in SIRT1 activity concomitant with the induction of R-loops was also detected during the propagation of these primary fibroblasts in culture. The pharmacological inhibition of SIRT1 also led to an increased abundance of R-loops, suggesting a causal relationship. R-loops were highly prevalent in cancer cells harboring mutated SIRT1 or the depletion of SIRT1 (Figure 7). The pharmacological inhibition of SIRT1 in all cell types and a genetic deactivation of SIRT1 in cancer cells triggered excess DNA synthesis via the initiation of DNA replication from dormant origins. In contrast, in non-transformed fibroblasts, aging-associated decline in SIRT1 activity was restricted to rDNA arrays. 

Since SIRT1 plays a role in dormant origin maintenance, it is logical to assume that the increased R-loop levels observed in cells with reduced SIRT1 activity, either throughout the genome or within the rDNA loci, reflect the consequence of increased replication origin activity. SIRT1 binds replication origins and maintains origin dormancy by deacetylating the replication complex component TOPBP1, thus preventing it from recruiting ATR and thereby disallowing an activating, ATR-mediated phosphorylation of the helicase component MCM2 on serine 108 [21]. This proposed mechanism is in line with the suggestion that R-loops reflect encounters of the transcription machinery with excess or disorderly replication [24,25,35,36,37]. Nevertheless, our observations do not rule out another mechanism whereby SIRT1 affects the formation of R-loops by modulating chromatin compaction and transcription. As SIRT1 can deacetylate a variety of substrates, including transcription factors and histones, it is possible that the deacetylation of another SIRT1 substrate can suppress transcriptional activity and prevent the formation of R-loops. It is also possible that SIRT1 plays a crucial role in resolving/blocking replication machinery bypass, which leads to the formation of post-replicative R-loops [38].

Interestingly, in cancer cells, we observed that SIRT1 inhibition, or the replacement of SIRT1 with an active site mutant, resulted in a higher induction of R-loops than a complete depletion of the SIRT1 protein. These observations suggest that the abundance of R-loops increases in the presence of an inactive form of SIRT1, consistent with a mechanism whereby the SIRT1 protein binds to a target on chromatin and R-loops are formed as the bound SIRT1 fails to catalyze deacetylation. This mechanism is consistent with a scenario whereby SIRT1 activity is required to remove an inhibitory acetylation from a protein that induces excess replication. In such a case, binding to an inactive form of SIRT1 will ensure that the substrate remains in an unacetylated form, whereas SIRT1 deficiency might allow other deacetylases to substitute for SIRT1. Additional mechanistic studies are required to decipher the exact molecular mechanism involved in the SIRT1-mediated suppression of initiation at dormant origins. 

Unlike in cancer cells, the reduction in SIRT1 activity in primary fibroblasts (by the direct inhibition of SIRT1 or as a consequence of chronological aging) was associated with some increase in the prevalence of R-loops, but not with a genome-wide activation of dormant origins. These observations are consistent with the reported deleterious consequences of limited over-replication in untransformed cells [39,40]. It is likely, therefore, that in untransformed cells, SIRT1-mediated origin dormancy is complemented by additional pathways that prevent excess replication or kill cells that undergo such replication [41,42]. The evolutionary development of mechanisms to prevent excess replication in untransformed cells and as a barrier to transformation helps to maintain genomic stability and is incapacitated in immortalized cells and cancer cells that endure high levels of replication stress [43,44,45].

The SIRT1 activity declined and the prevalence of R-loops rose with chronological aging and with the passage of cells in culture in primary untransformed fibroblasts. However, because we did not detect changes in the genome-wide utilization of dormant origins during chronological aging, the mechanistic basis for the increased prevalence of R-loops in those cells does not involve the genome-wide activation of excess replication. However, replication initiation rates increased, and dormant origins were activated at rDNA loci, consistent with a markedly enhanced prevalence of replication-associated R-loops. The activation of excess replication was observed in rDNA loci regardless of the cell transformation status, suggesting that replication initiation rates within the rDNA repeat arrays were responsive to the SIRT1 levels in all cells. The elevated response of rDNA loci to sirtuin levels is akin to the observed modulation of the stability of the rDNA repeats by SIR2, the yeast ortholog of SIRT1. Notably, yeast SIR2 suppresses the production of rDNA extrachromosomal circles, which accumulate in aging yeast cells [5] along with regulating the extent of yeast lifespan [46], as well as mediating transcriptional silencing [18,47]. The effect of SIR2, along with the nucleosome binding protein SIR3, on replication origin choice is achieved by modulating the distribution of replication initiation sites in heterochromatin and euchromatin, likely via alterations in histone acetylation levels, which, in turn, affect MCM loading [19,20,48]. A direct association between the extent of the initiation of DNA replication and rDNA duplication was shown in mice, as the abundance of MCM2, a component of the replicative helicase, correlates with rDNA copy number [49]. In all eukaryotes, the tandemly repeated and highly transcribed rDNA loci are very fragile as a result of replication stress. Distinct signaling cascades keep the duplication of select rDNA tandem repeats at specific times during the S-phase [27] under strict copy number control [27,50]. Hence, it is plausible that, in non-transformed cells, although the inhibition of SIRT1 activity does not lead to persistent excess replication in most loci, the rDNA loci manifest a distinct dependence on SIRT1 activity as a modulator of replication origin activation and suppressor of deleterious R-loops.

Previous research has established the putative role of sirtuins in protecting rDNA from excess replication and transcription in yeast, and studies in yeast models continue to investigate how SIR2 is involved in the initiation of DNA replication [18,19,47]. However, questions remain as to how mammalian SIRT1 regulates replication-associated processes. Our findings reveal a pivotal role for SIRT1 in the prevention and containment of R-loops, which might be deleterious given their mutagenic potential [40]. Importantly, in primary untransformed fibroblast cells, a reduction in SIRT1 activity did not result in a genome-wide activation of dormant origins, which may indicate a protective mechanism complemented by other factors that prevent excessive replication. The significance of such a mechanism in maintaining genomic stability is evident when contrasted with the replication stress observed in cancer cells, where safeguards against excess replication are compromised. Our study underscores the interplay between SIRT1, R-loops, and replication initiation, providing insights into the mechanisms governing genomic stability during aging. By elucidating the role of SIRT1 in safeguarding rDNA integrity, these findings may have broader implications for therapeutic approaches targeting SIRT1 in the context of aging and cancer.

## Data Availability

Raw read files, coverage tracks, and peak files for fibroblast sequencing data can be found at the NCBI Gene Expression Omnibus (GEO) under the accession code GSE247469. Cancer cell line data are available at GSE184353.

## Supplementary Materials

The following supporting information can be downloaded at: https://www.mdpi.com/article/10.3390/cells12222630/s1, Supplementary Figure S1: Sample Characterization of Normal Fibroblasts; Supplementary Figure S2: SIRT1 inhibition increases the prevalence of nucleolar and non-nucleolar R-loops in non-transformed fibroblasts and cancer cells; Supplementary Figure S3: Genomic association of R-loops with replication origins; Supplementary Figure S4: Chronological aging activates flexible origins; Supplementary Figure S5: In chronological aging, increased origin usage is specific to the rDNA array on all five chromosomes; Supplementary Figure S6: The transcription of ribosomal DNA is affected by the inactivity of SIRT1 or chronological aging.
