# Supplementary material for: SIRT1 Prevents R-Loops during Chronological Aging by Modulating DNA Replication at rDNA Loci
Source: Cells. 2023 Nov 15;12(22):2630. doi: 10.3390/cells12222630 (PMC10669956; doi:10.3390/cells12222630)
Supplement: Supplementary file 1 [file cells-12-02630-s001.zip › cells-2660244-supplementary.pdf]

A

Sample ID	Coriell ID	Age at sampling	PDL received	PDL at senescence
A29	AG04441B	29 yr	16	47
A44	AG13156	44 yr	7	37
B48	AG04446	48 yr	11	33
B63	AG12851	63 yr	5	18

B

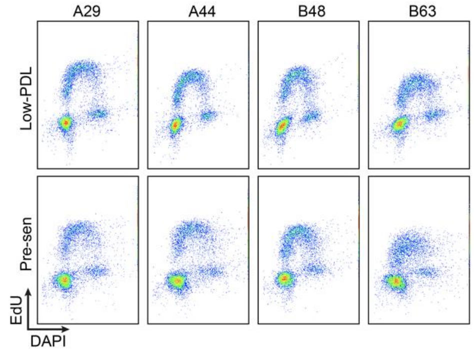

C

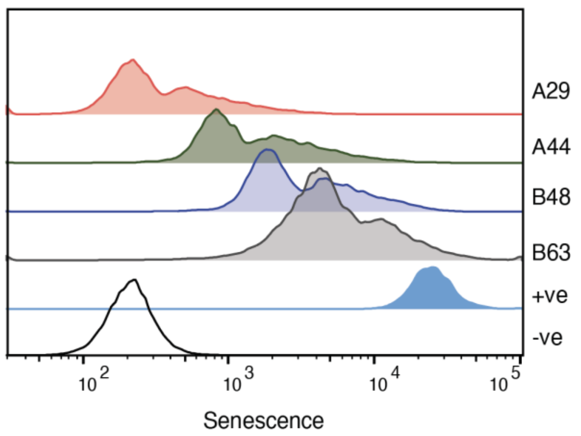

D

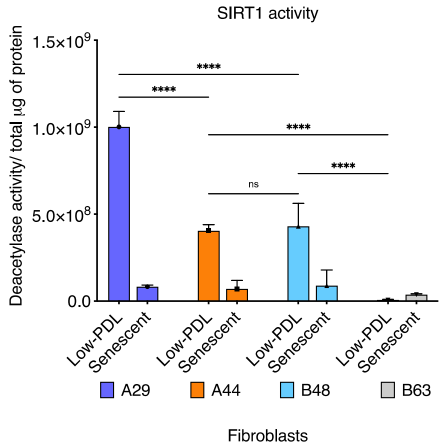

E

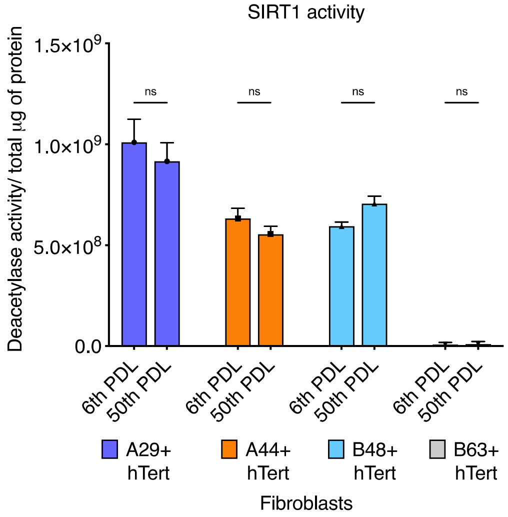

Supplementary Figure S1: Sample Characterization of Normal Fibroblasts. A: Normal human skin fibroblasts (Coriell depository) were obtained from individual A at age 29 and age 44, and from individual B at age 48 and age 63. **B:** Fibroblast cells from the early passage (low-PDL) and pre-senescent PDL (pre-sen) were incubated with EdU. Cell cycle progression and the fraction of (EdU-positive) replicating cells were measured by flow cytometry. **C:** The level of senescence across four fibroblast cells was quantified by flow cytometry using β-galactosidase levels . Unstained A29 (early passage) and 4-day-old culture of A29 irradiated at 10Gy were used as negative and positive controls. **D & E:** SIRT1 activity was markedly lower in samples taken from

individuals A and B at later points in chronological aging of fibroblasts (D) but not in immortalized fibroblasts at late passage PDL (E).

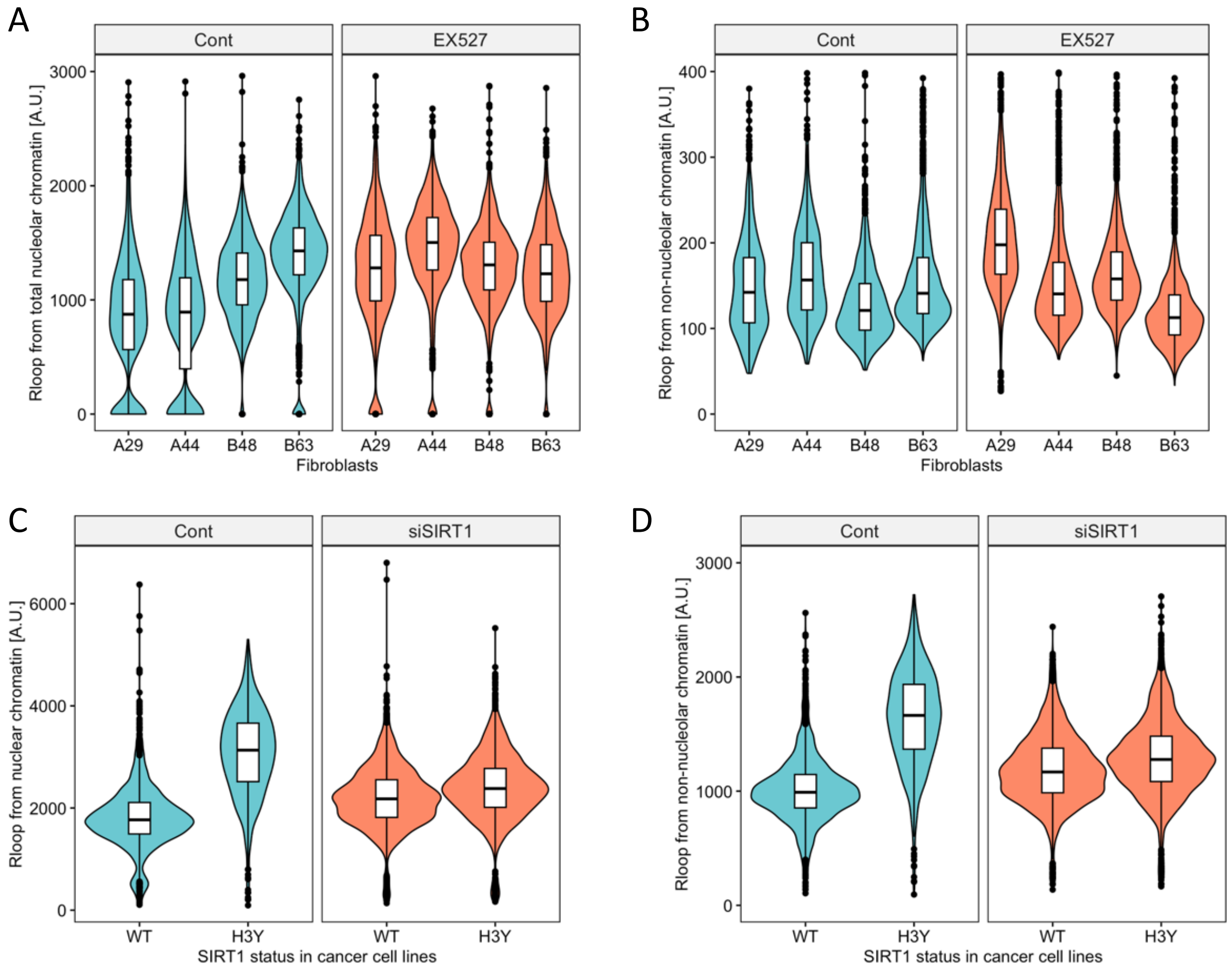

Supplementary figure S2: SIRT1 inhibition increases the prevalence of nucleolar and non-nucleolar R-loops in non-transformed fibroblasts and cancer cells.

A & B: Quantification of R-loops using immunofluorescence in total nuclear (A) and non-nucleolar (B) chromatin in chronological aging fibroblasts without and with chemical inhibition of SIRT1 . **C & D:** Prevalence of R-loops was measured in cells harboring WT- or H3Y-SIRT1 U2OS cells. Both WT and H3Y SIRT1 were depleted using siRNA. Quantification of R-loops using immunofluorescence as described in figure 1, total nucleolar (C) and non-nucleolar (D) chromatin plot displaying further increase in R-loop prevalence in cells with mutated SIRT1. siSIRT1 depletion increased R-loops in WT but not in H3Y, indicating role of SIRT1 activity in suppression of R-loop formation. Total nuclear and non-nucleolar R-loops were measured as described above. Unlike fibroblast cells, inhibition of SIRT1 increased the prevalence of R-loops in non-nucleolar chromatin in cancer cells.

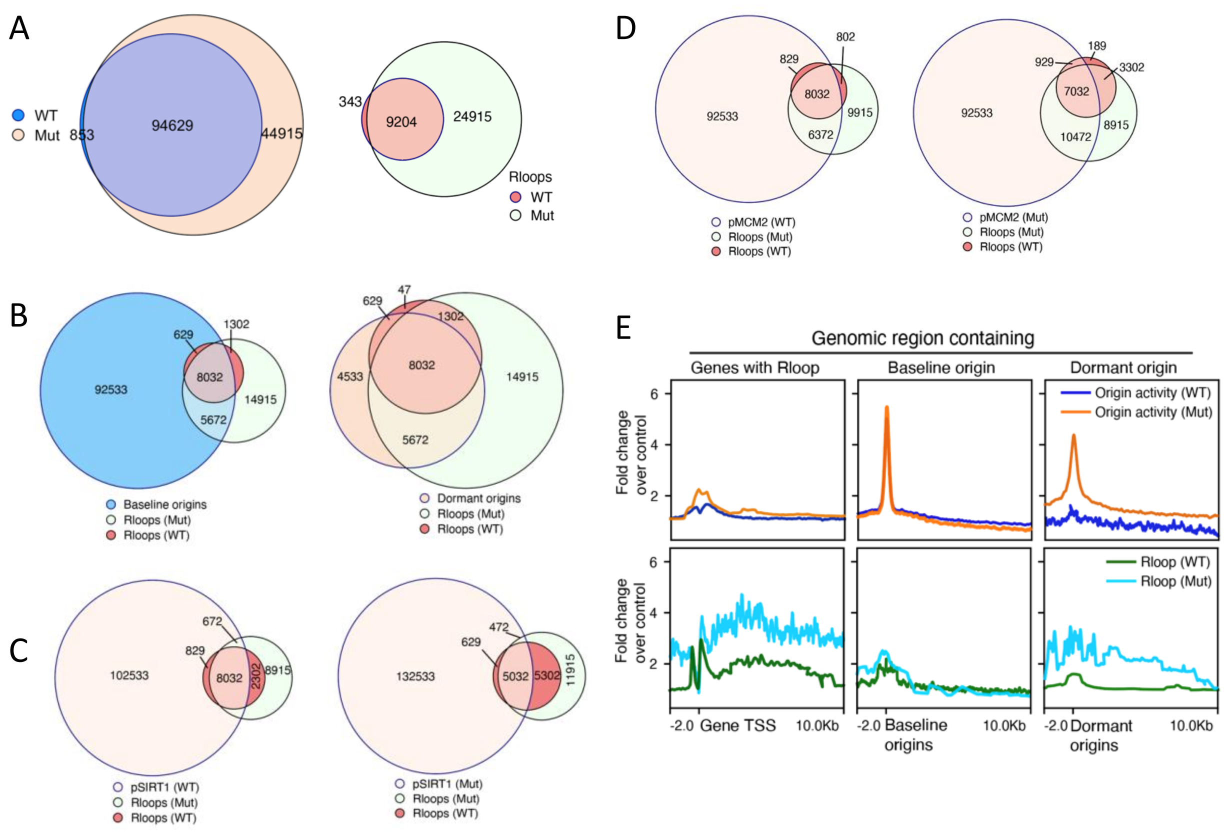

Supplementary figure 3: Genomic association of R-loops with replication origins.

A: Active SIRT1 prevents excessive origin activation and R-loop formation. Left, a Venn comparison between origins mapped from U2OS cells harboring WT- or Mut-SIRT1 with NS-seq . Right, Venn comparison between R-loops formed in U2OS cells harboring WT- or Mut-SIRT1.**B:** R-loops are prevalent in genomic locations containing dormant origins. Left, a Venn comparison between baseline origins location (all active origins from WT-SIRT1 cells) with R-loops mapped in WT and Mut-SIRT1 cells. Right, Venn comparison between genomic locations of dormant origins, R-loops from WT and Mut-SIRT1 expressing cells. **C & D:** Replication origin markers pSIRT1 and pMCM2 at dormant origins are associated with R-loop. (C) Left to right, pSIRT1 mapped from U2OS cells harboring WT- and Mut-SIRT1 compared with R-loops locations (D) Left to right, pMCM2 mapped from U2OS cells harboring WT- and Mut-SIRT1 compared with R-loops locations. **E:** From left to right, line plots summarizing the fold enrichment of origin activity (top) and R-loops formation (bottom) compared to respective controls at genes demonstrating R-loops, baseline, and dormant origins.

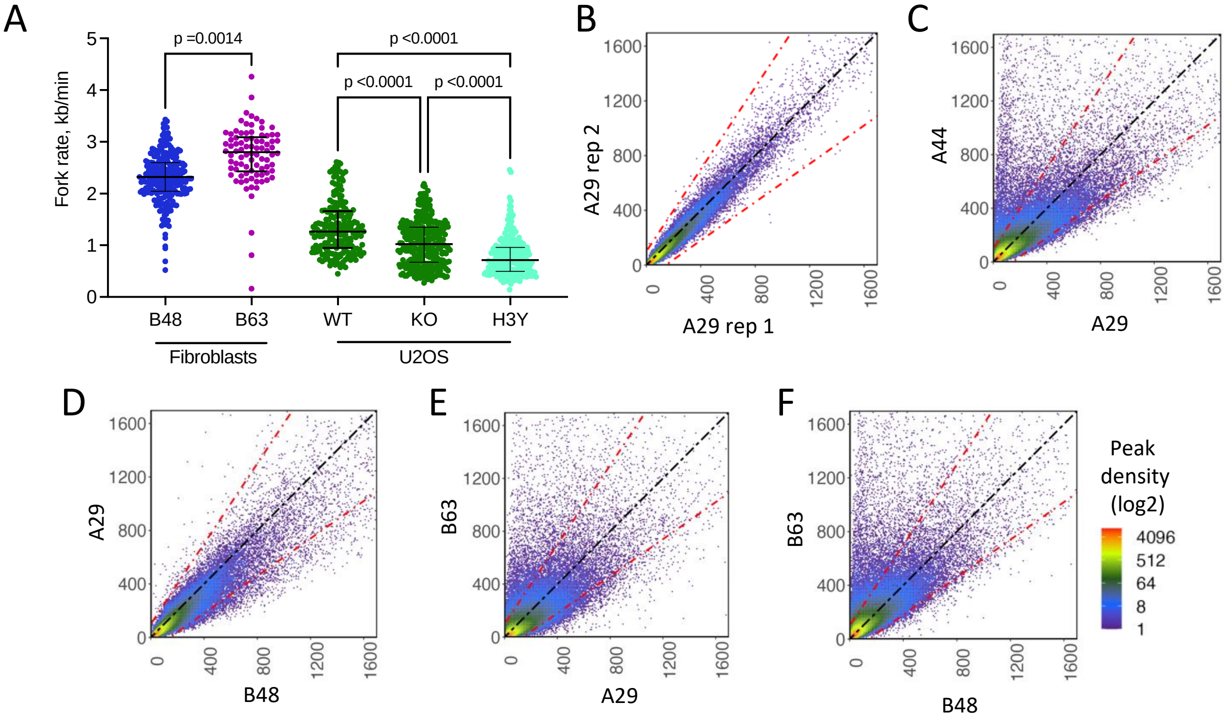

Supplementary figure 4: Chronological aging activates flexible origins.

A: Fork rates in DNA fibers from fibroblasts obtained from individual B at ages 48 and 63. and U2OS cells with active SIRT1 (WT), SIRT1 depleted (KO) or inactive SIRT1 (H3Y). **B:** A representative comparison between two replicates. **C-F:** Scatterplots showing origin activity over consensus origins mapped in all 4 fibroblast cells. Each dot represents a comparison between a mapped origin between two comparisons. Diagonal black dotted line indicates no change in origin activity while, red dotted line indicates 1.5 fold change in origin activity.

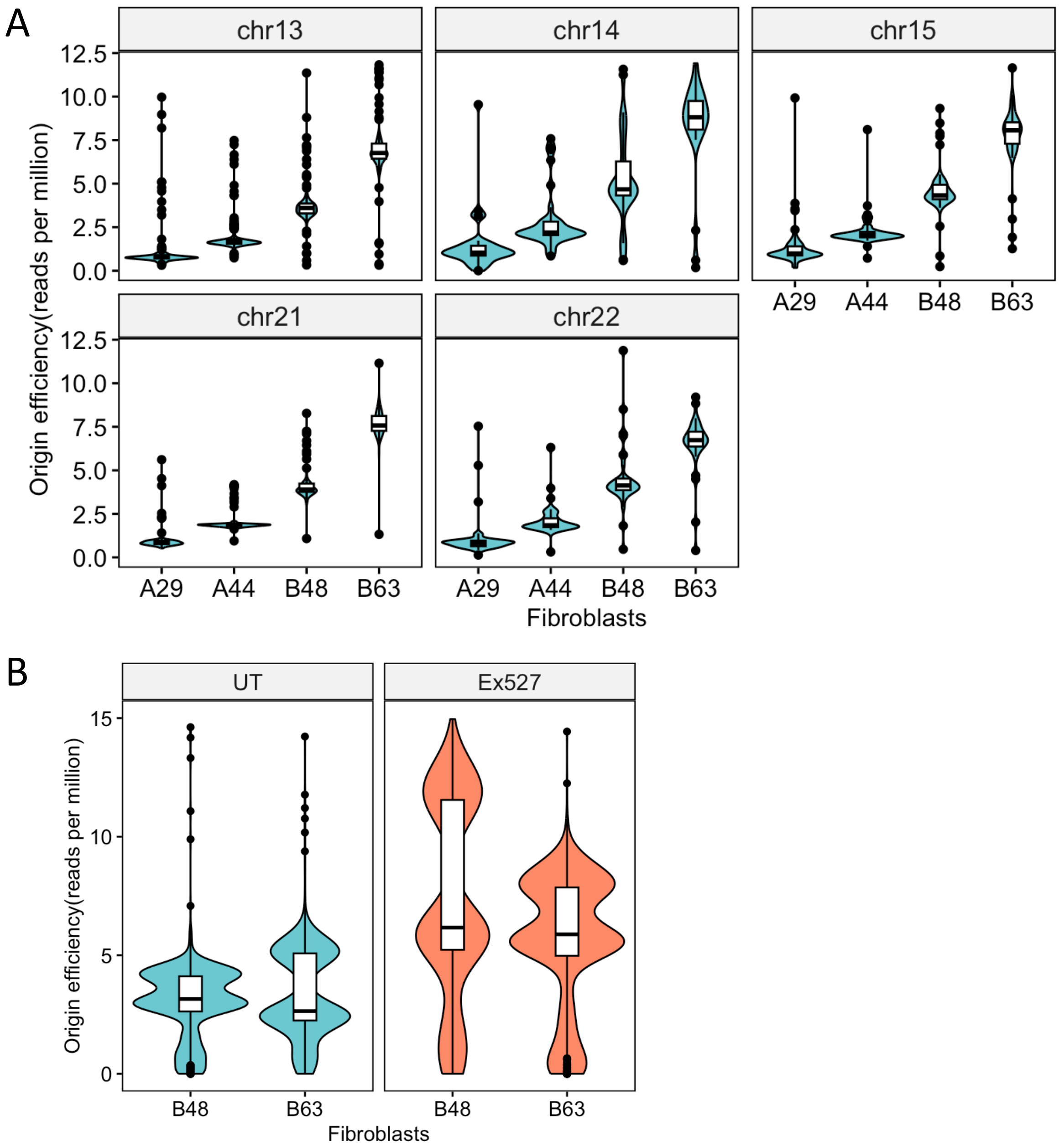

Supplementary figure S5: In chronological aging, increased origin usage is specific to the rDNA array on all five chromosomes.

A: The efficiency of origin replication was quantified from NS-seq of chronological age fibroblasts, and it was represented as a violin plot specific to each chromosome.

B: Dormant origins from the rDNA region are activated by chemical inhibition of SIRT1, in addition to chronological aging. The origin efficiency of chronological fibroblasts was quantified from NS-seq with and without EX527, and the results were plotted as violin plots.

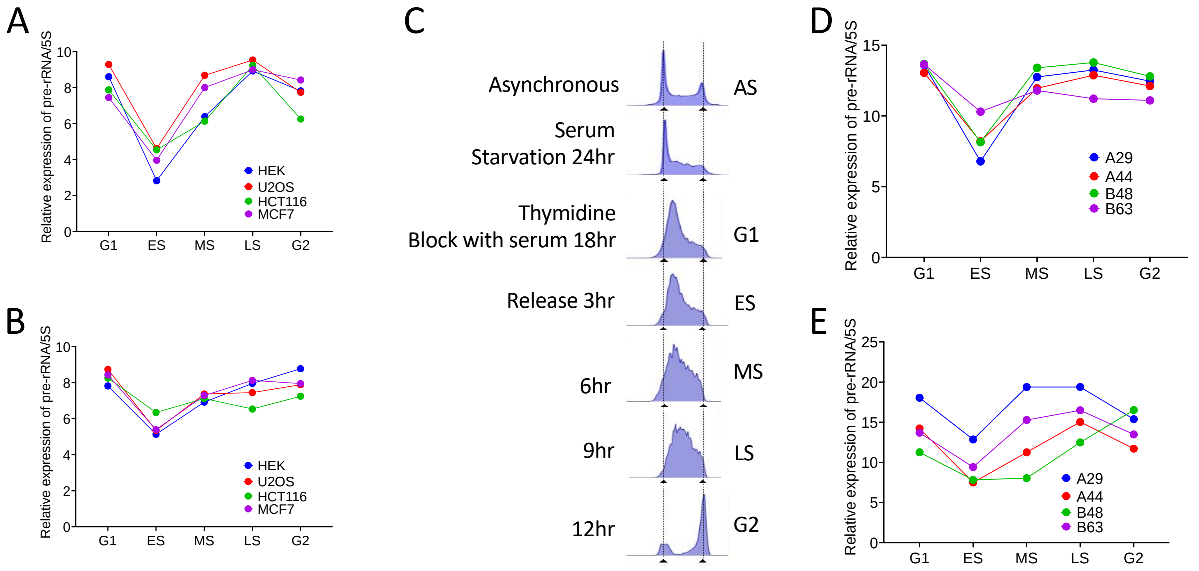

Supplementary Figure S6: The transcription of ribosomal DNA is affected by the inactivity of SIRT1 or chronological aging. A & B: Cancer cells with either wild-type (A) or knockout (B) SIRT1 status were synchronized in the G1/S phase using the double thymidine block technique. They were then released to obtain phase-specific cell cycles, and RNA was isolated. Quantitative PCR was performed to measure the transcript of 47S pre-rRNA. 5S rRNA was used as a normalization control. **C:** The cell cycle profiles were determined in fibroblast cells that were synchronized by serum starvation followed by double thymidine block. DNA content was measured by DAPI staining. **D & E:** Early passage (D) and pre-senescent (E) chronological fibroblasts were used to quantify 47S pre-rRNA transcripts following the scheme in C.
